# Analysis of costs and utilization of ambulance services in the ministry of health facilities, Malaysia

**DOI:** 10.1371/journal.pone.0276632

**Published:** 2022-11-04

**Authors:** Mohd Shahri Bahari, Farhana Aminuddin, Sivaraj Raman, Ainul Nadziha Mohd Hanafiah, Mohd Shaiful Jefri Mohd Nor Sham Kunusagaran, Nur Amalina Zaimi, Nor Zam Azihan Mohd Hassan, Ahmad Tajuddin Mohamad Nor

**Affiliations:** 1 Institute for Health Systems Research, Ministry of Health Malaysia, Shah Alam, Malaysia; 2 Hospital Tengku Ampuan Rahimah, Selangor, Malaysia; Central Queensland University, AUSTRALIA

## Abstract

**Background:**

Despite emergency ambulance services playing a pivotal role in accessibility to life-saving treatments in Malaysia, there are still numerous gaps in knowledge in terms of their utilization and cost. This leads to current policies on procurement, maintenance, and allocation being predicated on historical evidence and expert opinions. This study thus aims to analyse the cost and utilization of ambulance services in selected public health facilities in Malaysia.

**Methods:**

A cross-sectional study was employed involving 239 ambulances from selected hospitals and clinics. Ambulance service utilization was based on the number of trips, distance and duration of travel obtained from travel logbooks. A mixed top-down and activity-based costing approach was used to estimate the monthly cost of ambulance services. This constituted personnel, maintenance, fuel, overhead, consumables, ambulance, and medical equipment costs. The utilization and costs of ambulance services were further compared between settings and geographical locations.

**Results:**

The average total cost of ambulance services was MYR 11,410.44 (US$ 2,756.14) for hospitals and MYR 9,574.39 (US$ 2,312.65) for clinics, albeit not significantly different. Personnel cost was found to be the main contributor to the total cost, at around 44% and 42% in hospitals and clinics, respectively. There was however a significant difference in the total cost in terms of the type and age of ambulances, in addition to their location. In terms of service utilization, the median number of trips and duration of ambulance usage was significantly higher in clinics (31.88 trips and 58.58 hours) compared to hospitals (16.25 trips and 39.25 hours).

**Conclusions:**

The total cost of ambulance services was higher in hospitals compared to clinics, while its utilization showed a converse trend. The current findings evidence that despite the ambulance services being all under the MOH, their operating process and utilization reflected an inherent difference by setting.

## Introduction

Emergency Medical Service (EMS) functions to ensure a swift lifesaving treatment for those in need. It consists of a comprehensive network of agencies and trained personnel that coordinate emergency medical care, ranging from trauma centres to communication and transportation systems. One of the central elements of the realization of EMS is the availability of an emergency ambulance service (EAS). The recent COVID-19 pandemic and the sharp rise in demand worldwide have highlighted their importance while revealing gaps arising from a lack of resources [1, 2].

One of the critical pieces of information required for revamping the EAS is the availability of cost data [3]. However, a lack of cost data in the Asian countries including Malaysia limits the ability of policymakers to conduct necessary steps. This is important because the economic implication of expanding services is not only limited to ambulance purchases, but also its operational costs such as maintenance, overheads, and manpower. Additionally, as the duration of ambulance use is often extended beyond recommended periods due to resource limitations locally, its impact on service utilization and spending needs to be reviewed [4].

In Malaysia, the main provider of ambulance services is the Ministry of Health (MOH) through its public healthcare institutions [5]. According to the MOH Engineering Department, there was a total of 2,039 ambulances available nationwide up until May 2017. Out of these, 1,125 ambulances were located in hospitals and the rest were placed in health clinics. Interestingly, the operation and focus of EAS differ based on the type of facilities. Ambulances in hospitals are primarily designated for emergency and patient transfer, while those in clinics are more often multi-purposed. These facility-dependent services create a disparity in ambulance utilization which is often overlooked and crudely generalised.

An estimated MYR 170 million was set aside for annual ambulance purchases and operations under the National Budget for 2018. Additionally, the current reported bundled EAS charges offer limited value for economic evaluations of newer EAS strategies or exploration of their efficiencies. As such, similar to many developing countries, present policies on procurement, maintenance, and allocation of ambulances are often based on historical evidence and expert opinions [5]. Such predicaments hamper efforts by the policymakers to make the much-needed facility-specific improvements in the current time of uncertainties and economic downturn [4].

There is still a paucity of literature addressing the cost and utilization of ambulance services in Malaysia and other low to middle-income nations. This information is important because the economic implication of expanding services is not only limited to ambulance purchases, but also its operational costs such as maintenance, overheads, and manpower. Additionally, as the duration of ambulance use is often extended beyond recommended periods due to resource limitations locally, its impact on service utilization and spending needs to be reviewed [4]. This is because unwarranted expenditures in addition to possibilities of service disruptions may lead to diminished efficiency, reliability, and safety [5]. Thus, this study aimed to fill the gaps in knowledge on the utilization and cost of EAS in public healthcare hospitals and clinics, while addressing their differences.

## Material and methods

A cross-sectional study was conducted from the perspective of healthcare providers. Data on ambulance utilization and costs were collected between March and December 2019 from the emergency department of each selected public health facility under the MOH Malaysia.

### Study setting and sampling

The sampling frame included all public health facilities under MOH, the main provider of ambulance services in Malaysia. In 2019, a total of 144 hospitals and 698 health clinics in Malaysia were included in the sampling frame. A stratified random sampling method was used for the selection of the facilities. Malaysia is divided into two geographical regions; West Malaysia and East Malaysia. West Malaysia was further divided into four zones; Northern, East Coast, Central and Southern. To ensure that the findings of this cost analysis were representative, one state from each zone in West Malaysia and both states in East Malaysia were included in this study. Hospitals and clinics were also stratified to ensure equal representation of both the urban and rural areas. This consisted of a total of 76 health facilities (14 hospitals and 62 health clinics).

### Sample size estimation

The required sample size of ambulances to be included in this study was calculated via OpenEpi software using the annual distance travel of an ambulance between 2012 and 2016 [6], which fits the formula (N = Z_α_^2^S^2^/W^2^). Assuming a 95% confidence level, a desired total width (W) of confidence interval (5%) of 2043; a standard deviation (S) of 7036, and a standard normal deviate for α = Zα = 1.96, the total sample required was 182 ambulances. The final sample size with a 20% margin of loss was 228. All ambulances (type A, B or C) registered under the Engineering Services Division, MOH up to January 2019 were included. All ambulances (type A, B and C) registered under the Engineering Services Division, MOH up to January 2019 were included. Type C ambulances are fitted with only basic equipment such as an emergency and trauma kit, wheelchair, stretcher, and oxygen tank. On the other hand, type B and type A ambulances are supplied with additional equipment (type B: oxygen resuscitator, portable suction meter, automated external defibrillator, monitor, etc. and type A: portable automatic ventilator, glucometer, triage card, dead body management kit, etc.). Ambulances identified as beyond economic repair (BER) were excluded.

### Cost data collection

A mixed approach of top-down and activity-based costing was used to estimate the cost. Cost components were first identified based on the work process in EAS. They were categorized as either capital or recurrent cost. Capital costs are fixed, one-time expenses incurred on the purchase of an ambulance and medical equipment. Recurrent costs are the costs associated with ambulance utilization consisting of fuel, maintenance, personnel, overhead, and consumables. The frequency of activities and unit costs were obtained from travel records, expert opinions and available reports. Utilization data comprised of the number of trips made, distance travelled and duration of use.

### Valuation and sources

The cost components and data sources for the valuation process are defined in subsequent sections and summarized in Table 1. All cost components were totalled to generate the total cost per ambulance per month.

**Table 1:** Mixed top-down and activity-based costing (ABC) approach.

	Allocation factor	Measurement of costs	Data source
**Capital cost**			
Ambulance body	Total purchase price	Annualization factor with 5% discount rate (Life-year usage of 10 years)	Engineering Division, MOH & study sites
Medical equipment	Total purchase price	Annualization factor with 5% discount rate (Life-year usage of 5 years)	Engineering Division, MOH & study sites
**Recurrent cost**			
Personnel	Full-time equivalent	Monthly wage apportions with the duration of ambulance service	Study sites
Maintenance	Full-time equivalent	Monthly expenses on the scheduled service, repairs, and loss of use cost	Concession company
Fuel	Full-time equivalent	Monthly fuel cost recorded	Vehicle logbooks
Overhead	Floor space	Apportions of bills with floor space between emergency department and facilities	Study sites
Consumable	Floor space	Apportions of the consumable budget with floor space between the emergency department and facilities	Study sites

#### Capital cost

The capital cost consisted of expenditures for the ambulance and medical equipment procurement. The purchase costs were obtained from each study facility and verified by the Engineering Services Division, MOH as the main body for the ambulance procurement. The present value in 2019 was calculated using the annualization factor approach. A discount rate of 5% and a useful life of 10 years and 5 years were used for the ambulance and medical equipment, respectively. These useful life years were applied in conformity with the national asset useful life standards [7]. The equivalent annual cost of each asset was annualized using the discount rate with their respective useful life years.

#### Personnel

Personnel cost consists of the total salary of staff, inclusive of wages and allowances. The staff that are involved in ambulance service deliveries were first identified from discussions with the emergency physician and the head of the hospital emergency department. For emergency cases, this was set as one medical assistant and one driver, while for transfer or referral cases, this consisted of a doctor, a nurse and a driver. The time spent by each staff was identified by the duration of ambulance usage per month. The personnel cost was apportioned based on the working hours in delivering ambulance services.

#### Maintenance and fuel

This cost component consists of the scheduled service fees, repairs, parts replacement, and loss of use cost of each ambulance throughout the study period. Loss of use cost was calculated by multiplying the days of ambulance breakdown with MYR 60 (compensation for actual repair time per day) [8]. The total monthly costs of fuel purchased were obtained from a logbook of each ambulance and used in the calculation of the total cost of ambulance services.

#### Overhead

Overhead cost refers to expenses incurred such as electricity, water, telephone, and internet services. The monthly total bill was obtained from the finance department of each study facility. The overhead cost of the ambulance services was apportioned based on their floor space obtained from the engineering department. This was calculated by dividing the percentage of square meters of physical space occupied by the ambulance unit over the indoor area of the facilities.

#### Consumables

This included items that are disposable and require regular replacement, such as aprons, syringes, gauzes, cotton swabs, needles, intravenous lines, and oxygen tubes, among others. The cost of general consumables was obtained from the procurement section of the emergency department. As the number of patients transported was not available, floor space was used as the allocation basis to calculate the consumable cost.

#### Assumptions

Estimating the total cost of ambulance services required certain assumptions, where the number of personnel is constant for each ambulance trip. It was assumed that the number of personnel per trip was constant, while the number of transferred patients for each ambulance trip was one. Since there was no data available for a retired ambulance sale value, the scrap value for each ambulance was assumed to be zero.

By dividing the total cost of ambulance services with the desired utilization data collected, we were able to produce the average cost of ambulance utilization per month.

### Statistical analysis

The continuous variables were reported as mean (SD) or median (IQR) where appropriate while categorical data were reported in frequency and percentage. Cost data were tabulated in Microsoft Excel and merged with utilization data before being analysed using SPSS statistical software package version 26.0 (SPSS Inc., Chicago, Illinois, USA). The average cost of ambulance per utilization per month was also calculated by dividing the total cost of ambulance services with the utilization data collected. The difference in proportions was analysed using the Chi-square test. As utilization and cost data were highly skewed, the nonparametric Mann-Whitney U test and the Kruskal Wallis test were applied. The level of significance was set at p<0.05. Costs were reported in Malaysian Ringgit (MYR) and where possible presented in US$, by adjusting to 2019 levels using the exchange rate of US$ 1 to MYR 4.14 [9].

A one-way sensitivity analysis was performed to explore changes in total costs following variations in key parameters. The useful life of ambulance and medical equipment, discount rate, price of the ambulance and medical equipment, fuel cost and salary varied by 20% on either side of the base value. A tornado diagram was used to graphically represent the result.

### Ethics statement

This study was approved by the Medical Research and Ethics Committee (MREC), Ministry of Health Malaysia with reference number NMRR-18-2944-44909. Informed consent was not required by the Ethics Committee as the study did not involve human subjects.

## Results

### Ambulance characteristics

The majority of the ambulances in the hospitals (83.0%) and clinics (94.2%) were aged 10 years and below. Type B ambulances were the most commonly used in hospitals and clinics at 66.0 and 77.9%, respectively. About 57.0% of ambulances in the hospitals were dedicated for transfer, followed by emergency (30.7%). In clinics, ambulances were mostly multifunction (50.0%) and transfer (46.5%) (Table 2). The majority of the hospital ambulances were located in urban areas (69.9%), while for clinics, ambulances were located equally in urban (50.0%) and rural areas (50.0%). When compared by region, most of the ambulances were situated in hospitals and clinics in West Malaysia, with 64.7 and 65.1%, respectively.

**Table 2:** Characteristics of ambulance (n = 239).

Characteristics		Hospital (n = 153)		Clinic (n = 86)	
		n	%	n	%
Age of ambulance (years)	0–5	67	43.8%	44	51.2%
	6–10	60	39.2%	37	43.0%
	>10	26	17.0%	5	5.8%
Type of ambulance^a^	Type A	50	32.7%	10	11.6%
	Type B	101	66.0%	67	77.9%
	Type C	2	1.3%	9	10.5%
Function of ambulance	Emergency	47	30.7%	3	3.5%
	Multifunction	19	12.4%	43	50.0%
	Transfer	87	56.9%	40	46.5%
Location of ambulance	Urban	107	69.9%	43	50.0%
	Rural	46	30.1%	43	50.0%
Region	West Malaysia	99	64.7%	56	65.1%
	East Malaysia	54	35.3%	30	34.9%

^a^ Type of ambulance: Type A for the ambulance with Advanced Life-Support (ALS); Type B with Basic Life-Support (BLS); while Type C for others

### Ambulance utilization

Table 3 shows ambulance utilization for both hospitals and clinics. The number of trips was significantly higher in clinics (median = 31.88) compared to the hospital (median = 16.25), *U* = 4,800.0, p = 0.001. A Mann-Whitney test also revealed the duration of ambulance usage was statistically significant higher in clinics (median = 58.58 h) compared to hospitals (median = 39.25 h), *U* = 4,999.0, p = 0.002. On the other hand, no significant difference was found between distance of travel for ambulance in hospitals (median = 1,455.75 km) and ambulance in clinics (median = 1,768.75 km), *U* = 5,913.0, p = 0.194.

**Table 3:** Utilization of ambulance services (n = 239).

Ambulance utilization	Hospital (n = 153)				Clinic (n = 86)				p-value^a^
	mean	±SD	median	IQR	mean	±SD	median	IQR	
Distance of travel (km)	2,073.34	1,838.54	1,455.75	2,814.87	2,253.39	1,714.58	1,768.75	2,455.25	0.194
Number of trips	34.56	45.93	16.25	36.00	42.42	38.32	31.88	41.38	**0.001** *
Duration of usage (h)	61.98	60.26	39.25	96.06	91.07	82.62	58.58	98.93	**0.002** *

^a^ independent sample (Mann-Whitney U Test)

* p<0.05, is considered significant.

#### Ambulance utilization by age and location

**Fig 1:**
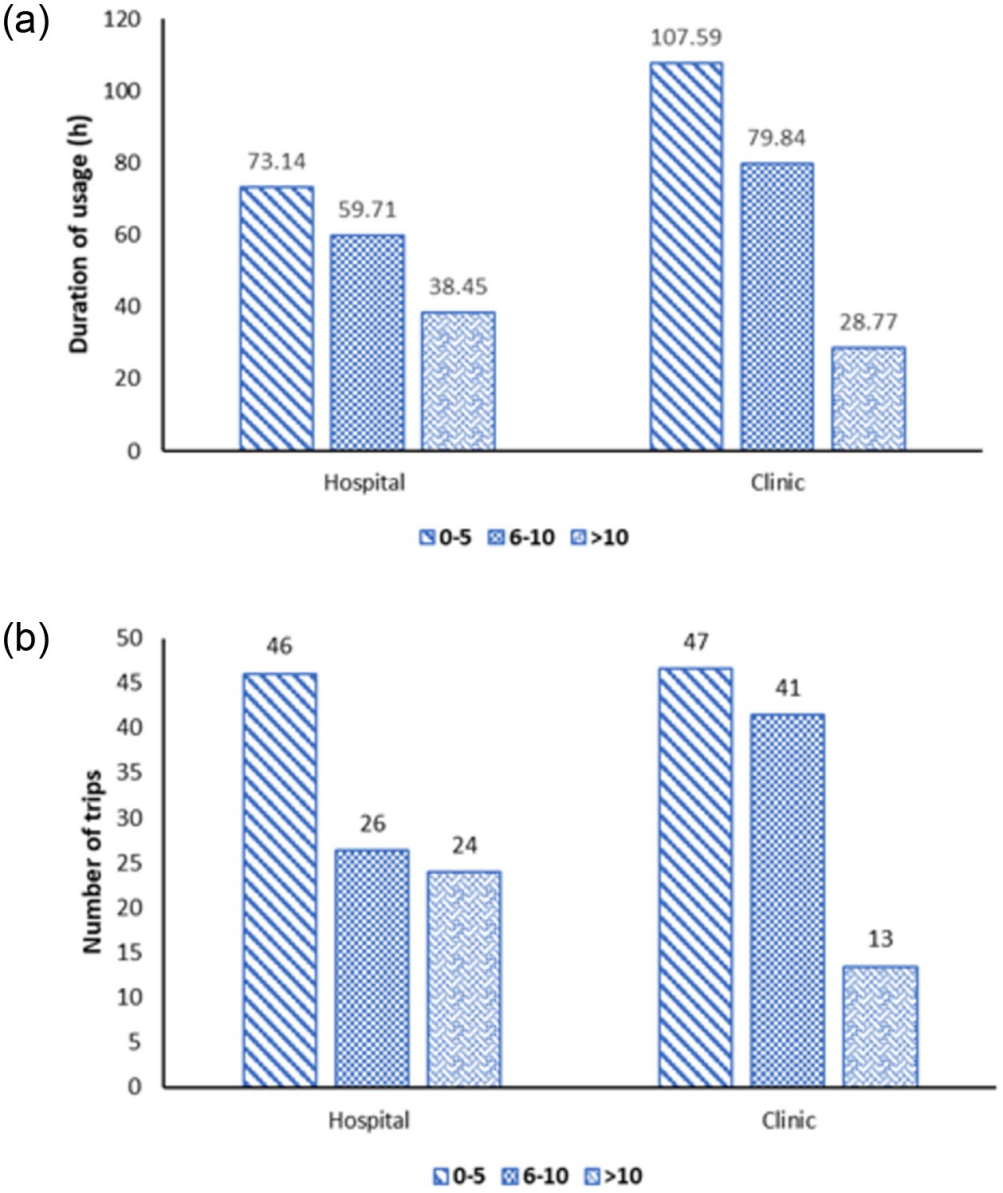
Ambulance utilization per month by age; A) Mean duration of usage by ambulance age; B) Mean number of trips by ambulance age.

There was a downward trend in the duration of usage (in hours, h) as the age of ambulances increased for both hospitals and clinics, as shown in Fig 1. For ambulances in hospitals, the highest duration of usage per month was among ambulances in the 0–5 years age group (73.14 h), followed by ambulances aged 6–10 years (59.71 h) and the lowest was for ambulances aged more than 10 years (38.45 h). Similarly, at the clinics, ambulances aged 0–5 years had the highest duration of usage per month (107.59 h) and those more than 10 years (28.77 h) had the lowest duration of usage. Hospital ambulances aged 0–5 years had a much higher number of trips (46 trips per month), compared to the hospital ambulance aged 6–10 years and those more than 10 years, with 26 and 24 trips per month, respectively.

**Fig 2:**
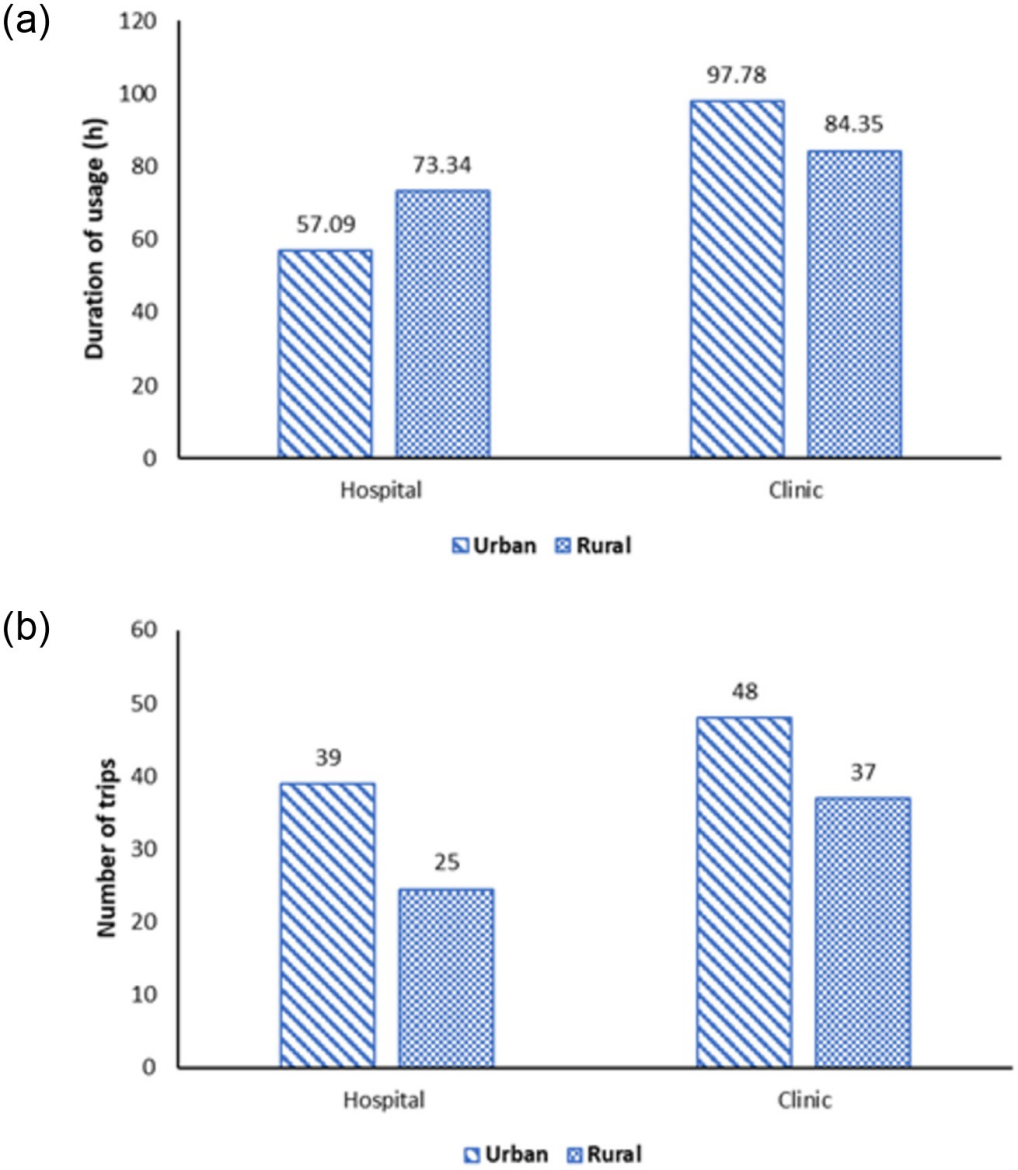
Ambulance utilization per month by ambulance location. A) Mean duration of usage by ambulance location; B) Mean number of trips by ambulance location.

Fig 2 shows ambulance usage per month by location. The duration of usage for hospital ambulances was higher in rural areas (73.34 hours) compared to urban areas (57.09 hours). On the contrary, the usage duration of clinic ambulances was higher in urban (97.78 hours) compared to rural areas (84.35 hours). The number of trips for ambulances in urban hospitals (39 trips per month) was higher compared to the ambulance in rural hospitals (25 trips per month). Similarly, the number of trips for the ambulance in urban clinics (48 trips per month) was higher compared to the ambulance in rural clinics (37 trips per month).

### Cost of ambulance services

Total cost and cost components of ambulance services for hospitals and clinics are shown in Table 4, while unit cost by utilization of ambulance is shown in Table 5. The average total cost of ambulance services in the hospital and clinics were MYR 11,410.44 ± 6,344.67 and MYR 9,574.39 ± 5,087.90, respectively (Table 4). Mann-Whitney U test showed no significant differences in monthly costs for ambulance service between hospitals (median = MYR 9,530.35) and clinics (median = MYR 9,047.38), *U* = 5609.0, p = 0.059.

**Table 4:** Cost of ambulance services (n = 239).

Cost components	Hospital (n = 153)					Clinic (n = 86)					p-value^a^
	mean	±SD	%	median	IQR	mean	±SD	%	median	IQR	
Personnel	5,003.22	4,892.90	43.9	3,116.02	7,996.42	3,983.39	4,123.93	41.7	2,999.58	4,155.63	0.448
Ambulance value	2,816.58	690.62	24.7	2,957.59	281.80	2,782.98	536.80	29.0	2,957.59	587.45	**0.040** *
Medical equipment value	1,694.13	1,322.97	14.8	1,576.18	2,814.03	1,386.09	935.83	14.5	1,796.65	2,259.28	0.548
Maintenance	996.42	1,404.47	8.7	625.00	1,160.00	339.47	751.35	3.5	82.49	437.38	**<0.001** *
Fuel	693.24	578.51	6.1	549.90	910.68	724.70	517.42	7.6	612.61	812.07	0.366
Overhead & consumable	206.86	94.07	1.8	174.10	43.39	357.77	183.44	3.7	309.56	440.88	**<0.001** *
Total	11,410.44	6,344.67	100.0	9,530.35	9,186.83	9,574.39	5,087.90	100.0	9,047.38	5,534.73	0.059

Note: All costs were reported in Malaysian Ringgit (MYR);

^a^ independent samples (Mann-Whitney U Test);

* p<0.05, is considered significant.

**Table 5:** Cost by ambulance utilization.

	Hospital	Clinic
Ambulance Cost per month (MYR)	11,410.44	9,574.39
**Utilization per month**		
Distance of travel (km)	2,073.34	2,253.39
Number of trips	34.56	42.42
Duration of usage (h)	61.98	91.07
**Unit Cost per utilization**		
Cost per distance of travel (MYR/km)	5.50	4.25
Cost per trip (MYR/trip)	330.15	225.68
Cost per duration of usage (MYR/h)	184.09	105.13

A disaggregation of the costs into the components (Table 4) showed that personnel cost was the main contributor to the total monthly cost for the ambulance in hospitals and clinics, accounting for 43.9% and 41.7%, respectively. This was followed by ambulance value and medical equipment value, with 24.7% and 14.8% for hospitals and 29.0% and 14.5% for clinics, respectively. Maintenance, fuel, and overhead costs contributed to the remaining 16.6% of total hospital ambulance cost and 14.8% of total clinic ambulance cost.

Mann-Whitney U test showed a significant difference of ambulance value between hospitals (median = MYR 2,957.59) and clinics (median = MYR 2,957.59), *U* = 5,549.0, p = 0.040. There was also a significant difference in maintenance cost for ambulances between hospitals and clinics *U* = 3,033.5, p < 0.001, with maintenance cost in hospitals (median = MYR 625.00) being significantly higher than in clinics (median = MYR 82.49). On the contrary, overhead and consumables cost was significantly higher in clinics (median = MYR 309.56) than the hospitals (median = MYR 174.10), *U* = 2,926.0, p < 0.001.

The unit costs of ambulance services by their utilization for hospitals were MYR 5.50 per km, MYR 330.15 per trip and MYR 184.09 per hour (Table 5). Whereas the unit costs of ambulance services by their utilization for clinics were MYR 4.25 per km, MYR 225.68 per trip and MYR 105.13 per hour.

### Factors affecting ambulance cost

Table 6 shows monthly ambulance costs for hospitals and clinics by ambulance characteristics. There was a significant difference in hospital ambulance cost between ambulance age groups H(2) = 38.8, p < 0.001, with the cost of ambulances aged more than 10 years (median = MYR 4,437.16) being significantly lower than those aged 0–5 years (median = MYR 12,281.55) and 6–10 years (median = MYR 8,334.59). There was also a significant difference in hospital ambulance costs between the types of ambulance H(2) = 20.967, p = 0.049, where the cost for Type A ambulance (median = MYR 12,167.95) were significantly higher compared to Type B (median = MYR 8,571.28) and Type C (median = MYR 2,141.25). The cost of hospital ambulance for transfer (median = MYR 8,934.74) was significantly lower compared to the multifunction (median = MYR 15,476.85) and emergency ambulances (median = MYR 10,340.78), H(2) = 6.032, p = 0.049. There was also a significant difference in the hospital ambulance cost between ambulance locations *U* = 1,961.0, p = 0.047, with the cost being higher in the rural (median = MYR 12,586.79) than in urban areas (median = MYR 9,109.99).

**Table 6:** Cost by ambulance characteristics (n = 239).

Characteristics		Hospital (n = 153)					Clinic (n = 86)				
		mean	±SD	median	IQR	p-value	mean	±SD	median	IQR	p-value
Age of ambulance (years)	0–5	14,349.95	6,340.93	12,281.55	9,928.48	**<0.001** ^a^ *	11,204.90	5,366.63	9,828.55	3,968.17	**<0.001** ^a^ *
	6–10	10,329.51	5,357.43	8,334.59	8,631.38		8,550.80	3,963.61	6,961.33	5,556.39	
	>10	6,329.99	4,249.76	4,437.16	5,094.19		2,800.51	1,673.17	1,859.39	3,174.85	
Type of ambulance	Type A	14,130.89	6,155.85	12,167.95	9,482.13	**<0.001** ^a^ *	11,486.39	3,702.27	11,265.41	5,060.83	**<0.001** ^a^ *
	Type B	10,247.23	5,986.12	8,571.28	9,296.23		9,946.13	5,210.23	9,265.63	5,535.86	
	Type C	2,141.25	82.69	2,141.25			4,682.53	1,737.36	5,103.20	2,673.65	
Function of ambulance	Emergency	12,766.72	6,761.51	10,340.78	8,797.11	**0.049** ^a^ *	8,707.09	3,512.53	8,190.52		0.126^a^
	Transfer	10,103.65	5,139.94	8,934.74	8,334.05		10,305.76	6,412.07	9,274.14	4,899.24	
	Multifunction	14,039.12	8,747.60	15,476.85	17,570.55		10,040.35	6,123.15	9,265.63	5,744.53	
Location of ambulance	Urban	10,891.62	6,539.60	9,109.99	9,182.47	**0.047** ^b^ *	11,410.65	5,510.41	10,702.08	5,936.82	**<0.001** ^b^ *
	Rural	12,617.25	5,753.66	12,586.79	10,433.90		7,738.14	3,888.55	6,961.33	4,965.03	

Note: All costs were reported in Malaysian Ringgit (MYR)

^a^ Kruskal Wallis Test

^b^ Mann Whitney U Test

* p<0.05, is considered significant.

Similarly, there was a significant difference in clinic ambulance cost between ambulance age groups, H(2) = 18.63, p < 0.001, where the cost of ambulances aged more than 10 years (median = MYR 1,859.39) was significantly lower than those aged 0–5 years (MYR 9,828.55) and 6–10 years (MYR 6,961.33). By types of ambulance, the cost of clinic ambulance for Type A (median = MYR 11,265.41) was significantly higher than Type B (median = MYR 9,265.63) and Type C (median = MYR 5,103.20), H(2) = 17.138, p < 0.001. There was also a significant difference in the cost of the clinic ambulance between ambulance locations, *U* = 507.0, p < 0.001. In the urban area, the cost of clinic ambulance service (median = MYR 10,702.08) was higher than in rural areas (median = MYR 6,961.33). There was no significant difference in clinic ambulance cost between the functions of ambulance H(2) = 4.148, p = 0.126.

### Sensitivity analysis

**Fig 3:**
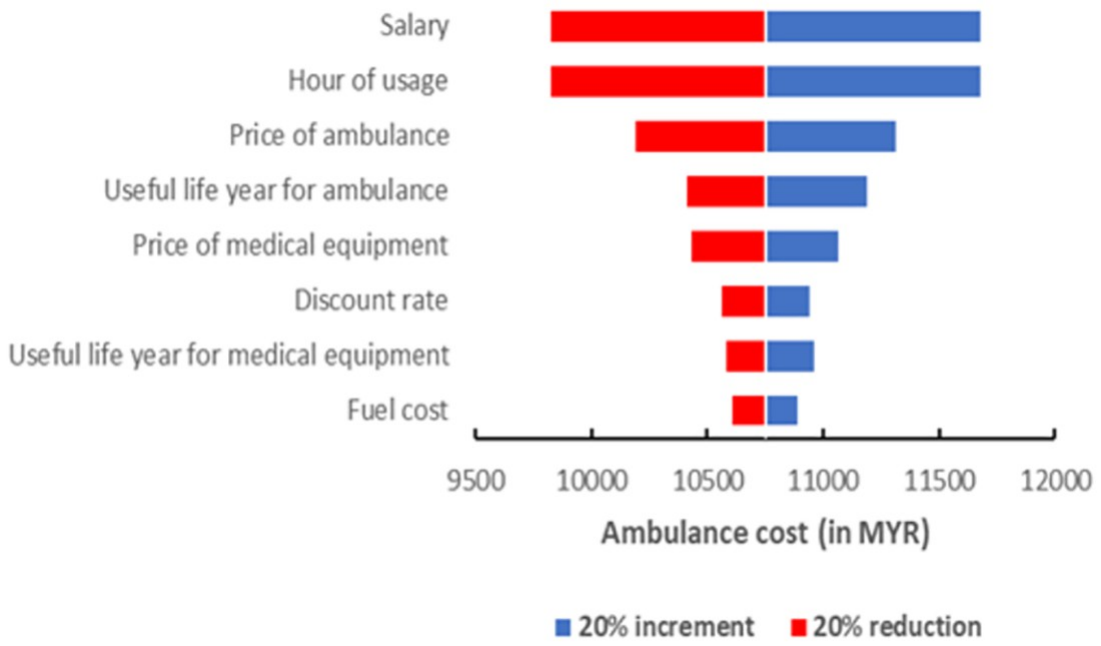
One-way sensitivity analysis tornado chart.

Fig 3 shows a tornado diagram of one-way sensitivity analysis for ambulance costs. Salary and duration of ambulance usage were the key drivers of the total cost. A 20% increment in personnel salary resulted in an increment in ambulance cost from MYR 10,749.77 to MYR 11,677.02, while a 20% reduction in personnel salary resulted in ambulance costs reduced to MYR 9,822.52. Similarly, a 20% increment in the duration of ambulance usage resulted in an increment in ambulance cost to MYR 11,677.02, while a 20% reduction led to the ambulance costs decreasing to MYR 9,822.52. Other important variables that impacted the total cost were the price of the ambulance, useful life years of the ambulance and price of medical equipment.

## Discussion

The pattern of ambulance utilization in Malaysia mirrored the differences in the healthcare services provided through its hospitals and health clinics, in addition to their geographical location. While such disparity was consistent across numerous studies around the world, these studies were primarily focused on the variation in the location of EMS rather than the differences in the operational and administration system [10–12]. Thus, the predictors reported were often based on the service demands within a similar EMS setting and workflow. For example, differences were shown to be contributed by the nature of the emergency, with incidences of trauma being higher in the urban population while complications associated with the elderly predominating in the rural population [13–15]. Additionally, demography-related variations can also be contributed by the perception of emergency, unbalanced distribution of pre-hospital EMS infrastructure, and ‘appropriateness’ of calls [11, 12, 15]. On the contrary, our findings demonstrated that despite the ambulance services being all under the umbrella of MOH, their utilization and process reflected an inherent difference by setting.

Our findings evidenced that the utilization of ambulances was higher in clinics compared to hospitals. This was largely due to the referral and emergency service pathway in the Malaysian public healthcare system which is guided by a clear delineation of care based on needs, availability of specialists, and demography [16]. Primary health clinics function as gatekeepers for access to limited specialist care in hospitals [17, 18]. Thus, clinics are predominantly involved in the transfer of patients to the nearest hospitals, as reflected by 96.5% of the ambulance function. This was also evidenced by the larger frequency of trips relative to hospital ambulances. The process of patient transfer is time-consuming, resulting in the average time of utilization of clinic ambulances being longer, despite having a comparable distance of travel with hospital ambulances [19].

Further analysis of ambulance utilization showed they were linked to their age and location. Ambulance age showed a critical trend of lower usage as the age increased. The disparity in usage was even larger in clinics, with the hours of use and trips falling by more than 72% for ambulances above the age of 10 compared with those less than five years. This could be due to the older ambulances being more prone to breakdown and unreliable for long-distance travel. While there is still a void in evidence reporting the impact of such incidences, two studies in India and sub-Saharan Africa concluded that a key factor in ensuring sustainable emergency services was to establish effective schemes for the maintenance of vehicles to ensure minimal downtime [20, 21].

The variation in costs may have also been contributed by their financing mechanism for ambulance maintenance and repair work. Contrary to hospitals where the costs are included as part of the ambulance acquisition concession agreement, the repairs and maintenance expenditures at clinics are covered under the total operating budget of respective clinics. In an already tight budget from increasing healthcare demands, this creates a challenge for continued planned preventive maintenance and urgent repair works, which may lead to longer downtime of clinic ambulances [20, 22].

The impact of the location of the ambulance service elucidated an interesting trend, where the association between utilization and duration of use was seen in rural areas for hospitals and urban areas for clinics only. Urban hospital ambulances had a lower duration of usage although making more trips compared to rural hospital ambulances. This may be because most urban hospitals are also state referral centres. Thus, while they had a larger demand, the distance of travel and the duration of use were minimal as they rarely required the transfer of patients to tertiary referral centres. On the contrary, rural hospital ambulances are required to travel further to access these tertiary referral centres. This was indirectly elucidated in the National Health and Morbidity Survey which showed that 48.6% of the urban population was within 10km to access in-patient care, compared to 72.6% in the rural area [23]. A contradicting pattern in clinics demonstrated that the utilization of ambulances in urban areas was probably driven by population density [10].

A disaggregation of costs into the components indicated that salaries were the biggest cost driver. This could be contributed by the large number of emergency personnel required for each ambulance trip, corresponding to a higher total personnel cost per hour. The finding was also comparable to the study conducted in India and Kenya which reported that personnel cost accounts for the major contributor to the total ambulance service cost with 36–50% and 49%, respectively [24, 25].

There were also significant variations in the ambulance value, maintenance, overhead and consumables costs between hospitals and clinics. While the median ambulance value was similar, the differences may have occurred statistically as the Mann-Whitney U test compares differences in terms of rank-sum. Ambulance maintenance, overhead and consumables costs were identified as more critical contributors to cost variations between hospitals and clinics. In hospitals, a higher cost was driven by a larger maintenance expenditure, compared to clinics. This could be due to the higher number of older ambulances located in the hospitals, thus requiring larger budgets to maintain them. On the contrary, ambulance overhead and consumables costs were significantly higher for clinics. This could be due to higher utilization (higher number of trips) and the apportionment method (floor space) used in this study.

It was expected that the ambulance age would play an important role in the total cost estimate. Perplexingly, the study found that the total cost decreased with the increase in ambulance age. The reason could be due to low utilisation of older ambulances (aged 10 and above), breakdown or being unreliable for long-distance travel. It was also possible that the higher number of unscheduled repairs and maintenance in the older ambulance was not captured within this short study period. The total cost of ambulance services was also influenced by the type of ambulance. Type A ambulance, occupied with advanced life support, was shown to cost the highest compared to type B and C. According to Chew and Chan [26], type A ambulances were available in larger cities while type B ambulances were more common in district hospitals or rural health facilities. Also, it is interesting to acknowledge the urban-rural location of ambulances, whereby the higher total cost of ambulance services was observed for hospitals located in rural areas and clinics located in urban areas. This could be a reflection of ambulance utilization (duration of usage) in both hospitals in rural areas and clinics in urban areas, wherein ambulances from rural hospitals and urban clinics may need to transfer patients to specialist or state hospitals that are located in urban areas.

The strength of this study lies in its novel findings of variation in cost and utilization between hospitals and clinics. Even though the mismatch between EMS utilization and cost was shown to be inherent to the provision of services in different settings, it highlighted a need to conduct further studies on the allocative efficiencies of EMS. As EMS is still an essential service regardless of its utilization, exploration of pooling resources through the cluster hospital programs or optimising trips may provide an option to maintain the equity of care while improving efficiencies. Additionally, the cost values obtained represent the much needed recent data on ambulance usage in a middle-income country, given that prior studies are often dated and focused on the cost of referral and service efficiency [24, 25, 27, 28]. Our findings can also be generalized to represent public healthcare in Malaysia as it included both hospitals and clinics.

There were however a few limitations in the present study. The actual burden of providing the EMS is expected to be larger than the value estimated. This is because the costing approach and study duration limited the comprehensiveness of data, especially in terms of ad-hoc maintenance costs. Furthermore, the commercial insurance value used for calculating the cost for loss of ambulance use may underestimate the actual cost from productivity losses. However, there is a possibility for the expenditures to be partly compensated if the scrap cost of ambulances were included. Secondly, floor space was used as the apportionment method for consumable cost allocation instead of headcounts. This may have further underestimated the consumptions of consumables. Lastly, the explanatory nature of the analysis shows only an unadjusted association between factors and the total costs estimated. It does not reflect the true cost determinants and the interactions between the cost components. A more in-depth and robust analysis is warranted to further ascertain such associations.

## Conclusions

This study elucidated that besides the demographical factors, the presence of aged ambulances may impact the optimization of service utilization. The findings provide further evidence on the importance of maintaining and improving the condition of ambulances to enable the provision of services. The obtained estimates on the duration of utilization and resource allocation can also be used for planning ambulance replacement schedules, scaling up procurement and ensuring improved use of financial resources. We hope that further cost-effectiveness analysis and efficiency studies can be conducted to consolidate these findings for policy-makers.
